# The role of serum tryptase in COVID-19 pathogenesis and its value as a prognostic marker: a single-center prospective cohort study

**DOI:** 10.3389/abp.2026.15936

**Published:** 2026-01-28

**Authors:** Alan Majeranowski, Damian Palus, Michał Hoffmann, Zbigniew Heleniak, Tomasz Stefaniak, Krzysztof Kuziemski

**Affiliations:** 1 Intercollegiate Faculty of Biotechnology, The University of Gdańsk, Gdańsk, Poland; 2 Department of Hypertension and Diabetology, Medical University in Gdańsk, Medical Faculty, Gdańsk, Poland; 3 University Clinical Centere, Medical University of Gdańsk, Gdańsk, Poland; 4 Department of Nephrology, Transplantology and Internal Medicine, Medical University in Gdańsk, Medical Faculty, Gdańsk, Poland; 5 Department of Quality in Healthcare, Medical University in Gdańsk, Medical Faculty, Gdańsk, Poland; 6 Department of Surgical Oncology, Transplant Surgery and General Surgery, Medical University in Gdańsk, Medical Faculty, Gdańsk, Poland; 7 Department of Pulmonology, School of Public Health, Collegium Medicum, University of Warmia and Mazury in Olsztyn, Olsztyn, Poland

**Keywords:** COVID-19, inflammation, mast cells, prognostic biomarker, tryptase

## Abstract

**Background:**

Dysregulated inflammation is central to COVID-19 pathogenesis. Mast cells (MCs) and their protease tryptase are implicated in tissue injury and vascular dysfunction, but the prognostic value of circulating tryptase in COVID-19 remains uncertain.

**Methods:**

We conducted a prospective cohort study including 82 patients with laboratory-confirmed COVID-19 admitted between January and March 2021. On admission, serum tryptase, C-reactive protein (CRP), procalcitonin (PCT), lactate dehydrogenase (LDH), and lymphocyte counts were measured. The objective of this study was to assess whether serum tryptase levels measured upon hospital admission are associated with COVID-19 severity and in-hospital mortality. Statistical analyses comprised t-tests, Mann–Whitney U tests, χ^2^ tests, receiver operating characteristic (ROC) curve analysis, and logistic regression.

**Results:**

Serum tryptase levels at admission did not differ significantly between patients requiring oxygen and those who did not (mean 5.45 vs. 4.97 μg/L; p = 0.2906) or between survivors and non-survivors (mean 5.24 vs. 5.60 μg/L; p = 0.6486). ROC analysis confirmed limited prognostic performance for tryptase regarding oxygen requirement (AUC = 0.580; p = 0.2972) and mortality (AUC = 0.538; p = 0.6103). By contrast, CRP, PCT, and LDH correlated strongly with disease severity. Elevated PCT (p = 0.0016) and LDH (p = 0.0360) were significantly associated with mortality. Logistic regression showed no independent association between tryptase and adverse outcomes.

**Conclusion:**

In this prospective cohort, serum tryptase measured at admission was not associated with COVID-19 severity or mortality, suggesting limited utility as a prognostic biomarker. Established markers, particularly PCT and LDH, outperformed tryptase in predicting adverse clinical outcomes. The negative findings are clinically relevant as they demonstrate that tryptase does not contribute to prognostic risk stratification in COVID-19, and its measurement does not provide added value beyond standard inflammatory biomarkers.

## Introduction

Severe acute respiratory syndrome coronavirus 2 (SARS-CoV-2) unleashes a multilayered inflammatory response in which epithelial injury, endothelial dysfunction, and microvascular thrombosis drive hypoxemic respiratory failure and multi-organ complications. Autopsy studies early in the pandemic established widespread pulmonary microangiopathy and intussusceptive angiogenesis as pathologic hallmarks of fatal COVID-19, underscoring the centrality of vascular–immune cross-talk to disease progression (Ackermann et al., 2020).

Among innate sentinels poised at the airway and perivascular interfaces, mast cells (MCs) have emerged as plausible amplifiers of COVID-19 pathology. Human and experimental data show MC activation in SARS-CoV-2 infection: lung tissue from severe/fatal cases contains increased tryptase+/chymase + MCs with ultrastructural evidence of degranulation, and circulating MC proteases (notably chymase and CPA3) are elevated and track with clinical severity (Tan et al., 2023; Krysko et al., 2022; Wismans et al., 2023). Tan et al. demonstrated in animal model that serum chymase, more than tryptase, associated with severe disease and lung pathology, whereas Krysko et al. reported that multiple MC proteases, including tryptase, rose with severity (Tan et al., 2023; Krysko et al.).

From a molecular perspective, tryptase—the dominant granule serine protease of human MCs—has several actions pertinent to COVID-19 pathobiology. Tryptase cleaves and activates protease-activated receptor-2 (PAR-2) on epithelial and endothelial cells, promoting pro-inflammatory cytokine release (e.g., IL-6, IL-8), increased permeability, and bronchoconstriction (Molino et al., 1997; Cicala and Cirino, 2002; Itoh et al., 2005). It can also contribute to matrix remodeling and fibrosis through proteolysis of extracellular components and PAR-2–dependent signaling (Cicala and Cirino, 2002; Itoh et al., 2005). With respect to coagulation, tryptase–heparin complexes can modulate clot architecture and fibrinolysis, and experimental systems indicate effects on thrombus biology that dovetail with the immunothrombotic features of COVID-19 (Prieto-García et al., 2012; Samoszuk et al., 2003).

Yet the clinical performance of serum tryptase as a biomarker in COVID-19 has been inconsistent across studies (Krysko et al., 2022; Wismans et al., 2023). Two features likely contribute. First, sampling kinetics: acute tryptase peaks are short-lived (biological half-life ∼2 h), so blood drawn outside a narrow window may miss elevations (Valent et al., 2023). Second, compartmentalization: MC activation may be tissue-restricted (airway/lung), with minimal “spill-over” to blood—consistent with stronger signals for chymase/CPA3 than for tryptase in several cohorts (Tan et al., 2023; Krysko et al., 2022; Wismans et al., 2023).

Tryptase is a mast cell mediator well known for its role in allergic reactions, but its role in viral infections remains unclear. To place this in context, we compared tryptase with biomarkers already established in COVID-19 prognosis. A large systematic review and meta-analysis showed that inflammatory markers such as CRP, PCT, and LDH are consistently elevated in patients with severe COVID-19 and are associated with higher mortality (Hariyanto et al., 2020). In addition, Henry and colleagues, in an early pooled analysis, reported that elevated LDH and CRP were among the strongest laboratory predictors of severe and fatal disease (Henry et al., 2020). More recently, a meta-analysis focused specifically on procalcitonin confirmed its strong prognostic value, with pooled sensitivity and specificity values indicating meaningful predictive accuracy for both severity and mortality (Kumar et al., 2022). Against this backdrop, our study sought to evaluate whether tryptase, as a mast cell–derived biomarker, might provide complementary prognostic information to these established markers. In this study, we asked whether serum tryptase measured upon hospital admission could serve as a prognostic biomarker in COVID-19, specifically whether it could identify patients at higher risk of requiring oxygen therapy or in-hospital death.

## Materials and methods

### Study design and participants

A prospective cohort study was conducted in the COVID-19-dedicated wards at the University Clinical Center in Gdańsk, Poland. Patients were eligible for inclusion if they met the following criteria: (1) age ≥18 years, (2) confirmed SARS-CoV-2 infection via nasopharyngeal PCR testing, (3) provision of informed and voluntary consent to participate. Exclusion criteria included: (1) confirmed diseases associated with elevated baseline tryptase levels (chronic kidney disease, malignancies, acute coronary syndromes, asthma, anaphylaxis, mastocytosis), (2) refusal or withdrawal of consent during the study.

Patients were divided into two cohorts: the first included those with respiratory failure requiring oxygen therapy, via nasal cannula, face mask, high-flow nasal oxygen therapy (HFNOT), or mechanical ventilation, and the second comprised those who did not require oxygen therapy. After providing consent, patients underwent laboratory tests (serum tryptase, LDH activity, CRP, PCT, complete blood count) within 0–3 days of admission. The reference intervals were as follows: tryptase <11.4 μg/L, C-reactive protein (CRP) <5 mg/L, procalcitonin (PCT) < 0.05 ng/mL, lactate dehydrogenase (LDH) 120–246 U/L, and lymphocyte count 1.0–4.5 × 10^9^/L. Serum tryptase was measured using a fluoroenzyme immunoassay (FEIA) on the ImmunoCAP platform (Thermo Fisher Scientific, Uppsala, Sweden). The analytical measurement range was 1–200 μg/L, with an analytical sensitivity of approximately 1 μg/L. Samples were stored at 2 °C–8 °C for up to 2 days. All analyses were performed according to the manufacturer’s protocol. According to the World Health Organization, body mass index (BMI) categories are defined as underweight (<18.5 kg/m^2^), normal weight (18.5–24.9 kg/m^2^), overweight (25.0–29.9 kg/m^2^), obesity class I (30.0–34.9 kg/m^2^), obesity class II (35.0–39.9 kg/m^2^), and obesity class III (≥40.0 kg/m^2^).

Clinical assessments included age, sex, comorbidities, body mass index (BMI) and oxygen dependency status. Follow-up during hospitalization involved daily assessments of oxygen dependency and occurrence of COVID-19 complications.

The primary objective of this study was to assess whether serum tryptase levels measured upon hospital admission are associated with COVID-19 severity and in-hospital mortality. Accordingly, the primary endpoint was the relationship between tryptase concentration and mortality, while secondary endpoints included associations with oxygen dependency, occurrence of COVID-19 complications, and length of hospitalization. Data collection started on 1 January 2021, and ended on 31 March 2021. An anonymized database was created using Excel.

### Statistical methods

The statistical analyses have been performed using the StatSoft statistical suite. Inc. (2014). STATISTICA (data analysis software system). version 12.0^1^ and Excel. The quantitive variables were characterized by the arithmetic mean of standard deviation or median or max/min (range) and 95% confidence interval. The qualitative variables were presented with the use of count and percentage. In order to check if a quantitive variable derives from a population of normal distribution the W Shapiro-Wilk test has been used. Whereas to prove the hypotheses on homogeneity of variances Leven (Brown-Forsythe) test has been utilized. Statistical significance of differences between two groups (unpaired variables model) was processed with the t-Student test or the U Mann-Withney test. The significance of difference between more than two groups were assessed with the F test (ANOVA) or Kruskal-Wallis. In the case of statistically significant differences between two groups *post hoc* tests were utilized (Tukey test for F or Dunn for Kruskal-Wallis). In the case of a two-pairs variables model, the t-Student or the Wilcoxon signed-rank (if t-Student conditions are not fulfilled or for variables measured on an ordinal scale) test was utilized. The significance of difference between more than two variables in the paired variables model has been checked by analysis of variance with repeated measurements or by Friedman test. Chi-squared tests for independence were used for qualitative variables. In order to determine dependence, strength and direction between variables, correlation analysis was used by determining the Pearson or Spearman’s correlation coefficients. In all the calculations the statistical significance level of p = 0.05 has been used.

## Results

A total of 82 patients hospitalized with confirmed SARS-CoV-2 infection were enrolled in the study. Of these, 20 patients (24.4%) were classified as oxygen-independent, while 61 patients (74.4%) required oxygen therapy during hospitalization and were categorized as oxygen-dependent. Subgroup analyses were performed by stratifying patients according to oxygen dependency and survival status, which reflect clinically meaningful severity categories. Additional subgrouping into WHO-defined severity classes was not performed due to limited sample size.

Statistical analysis revealed no significant differences between the two groups regarding age, sex, body mass index (BMI), or hospitalization duration. Detailed baseline characteristics are presented in Table 1.

**TABLE 1:** Baseline characteristics of the study group and oxygen-independent and oxygen-dependent patients regarding age, gender, and hospitalization duration.

Variable	Oxygen-independent (n = 21)	Oxygen-dependent (n = 61)	COVID-19 (n = 82)	*P*-value
Age (years)	​	​	​	0.1675^a^
Mean (SD)	61.4 (21.2)	68.7 (15.2)	66.9 (17.0)	​
Range	21.0–96.0	35.0–99.0	21.0–99.0	​
Median	62.0	68.0	68.0	​
95%CI	[51.5; 71.3]	[64.8; 72.6]	[63.1; 70.6]	​
Sex	​	​	​	0.4756^b^
Female	9 (45.0%)	22 (36.1%)	31 (38.3%)	​
Male	11 (55.0%)	39 (63.9%)	50 (61.7%)	​
Hospitalization (days)	​	​	​	0.5469^c^
Mean (SD)	13.1 (6.3)	15.4 (10.5)	14.9 (9.7)	​
Range	2.1–25.2	2.0–66.8	2.0–66.8	​
Median	12.8	13.3	13.0	​
95%CI	[10.2; 16.1]	[12.7; 18.1]	[12.7; 17.0]	​
BMI (kg/m^2^)	​	​	​	0.1543^c^
<18.5	0 (0.0%)	3 (4.9%)	3 (3.8%)	​
18.5–24.99	9 (47.4%)	11 (18.0%)	20 (25.0%)	​
25–29.99	5 (26.3%)	23 (37.7%)	28 (35.0%)	​
30–34.99	3 (15.8%)	15 (24.6%)	18 (22.5%)	​
35–39.99	1 (5.3%)	7 (11.5%)	8 (10.0%)	​
>40	1 (5.3%)	2 (3.3%)	3 (3.8%)	​

^a^ t-Student.

^b^ Chi2.

^c^ U Mann-Whitney.

BMI, body mass index; CI, confidence interval; SD, standard deviation.

P-values were calculated using the Student’s t-test or Mann–Whitney U test for continuous variables and the Chi^2^ test for categorical variables.

The two groups showed statistically significant differences in CRP, PCT, and LDH levels, with higher values observed in the oxygen-dependent group. No significant differences were found between groups regarding tryptase and lymphocyte counts. Detailed laboratory parameters are presented in Table 2.

**TABLE 2:** Comparison of laboratory parameters between oxygen-independent and oxygen-dependent COVID-19 patients.

Laboratory parameter	Oxygen-independent (n = 21)	Oxygen-dependent (n = 61)	COVID-19 (n = 82)	*P*-value
Tryptase (µg/L)	​	​	​	0.2906^a^
Mean (SD)	4.97 (5.00)	5.45 (4.42)	5.33 (4.55)	​
Range	1.00–24.10	1.28–27.20	1.00–27.20	​
Median	3.57	4.26	4.15	​
95%CI	[2.63; 7.31]	[4.32; 6.58]	[4.33; 6.34]	​
Tryptase category	​	​	​	0.8387^b^
Norm	18 (94.7%)	57 (93.4%)	75 (93.8%)	​
Above normal	1 (5.3%)	4 (6.6%)	5 (6.3%)	​
CRP (mg/L)	​	​	​	**0.0006** ^a^
Mean (SD)	37.82 (48.35)	103.11 (91.31)	86.99 (87.24)	​
Range	1.23–194.92	0.62–382.62	0.62–382.62	​
Median	17.45	76.38	56.20	​
95%CI	[15.19; 60.45]	[79.72; 126.50]	[67.70; 106.28]	​
PCT (ng/mL)	​	​	​	**0.0253** ^a^
Mean (SD)	0.09 (0.06)	2.16 (7.65)	1.66 (6.71)	​
Range	0.02–0.19	0.02–37.58	0.02–37.58	​
Median	0.07	0.13	0.12	​
95%CI	[0.05; 0.12]	[-0.36; 4.67]	[-0.25; 3.56]	​
LDH (U/L)	​	​	​	**0.0144** ^a^
Mean (SD)	274.4 (147.0)	425.8 (281.6)	390.0 (263.1)	​
Range	162.0–674.0	105.0–1,546.0	105.0–1,546.0	​
Median	210.0	320.5	301.0	​
95%CI	[185.6; 363.2]	[338.0; 513.5]	[318.9; 461.1]	​
Lymphocytes (×10^9^/L)	​	​	​	0.1468^a^
Mean (SD)	1.22 (0.67)	0.98 (0.42)	1.04 (0.50)	​
Range	0.39–3.31	0.30–2.94	0.30–3.31	​
Median	1.10	0.91	0.98	​
95%CI	[0.90; 1.54]	[0.87; 1.09]	[0.93; 1.15]	​

a U Mann-Whitney.

b Chi2.

c t-Student.

CRP–C-reactive protein; PCT, procalcitonin; LDH, lactate dehydrogenase; CI, confidence interval; SD, standard deviation. P-values were calculated using the Student’s t-test or Mann–Whitney U test as appropriate.

Bold values indicate statistically significant results (p < 0.05).

Patients who died were significantly older compared to survivors (mean 77.87 vs. 64.64 years, p = 0.0057). No statistically significant differences were observed between the groups regarding serum tryptase or CRP concentrations. However, deceased patients demonstrated significantly higher PCT levels (mean 3.08 vs. 1.23 ng/mL, p = 0.0016) and elevated LDH activity (mean 563.60 vs. 347.07 U/L, p = 0.0360). Detailed comparisons of demographic, biochemical, and hematological parameters are provided in Table 3.

**TABLE 3:** Comparison of demographic and laboratory parameters between surviving and deceased COVID-19 patients.

Variable	Survival (n = 67)	Death (n = 15)	*P*-value
Age (years)	​	​	0.0057^b^
Mean (SD)	64.64 (16.79)	77.87 (13.60)	​
Range	21.00–99.00	47.00–98.00	​
Median	67.00	76.00	​
95%CI	[60.55; 68.74]	[70.33; 85.40]	​
Tryptase (µg/L)	​	​	0.6486^c^
Mean (SD)	5.24 (4.49)	5.60 (4.82)	​
Range	1.00–27.20	1.28–21.90	​
Median	3.99	4.45	​
95%CI	[4.14; 6.33]	[2.93; 8.27]	​
CRP (mg/L)	​	​	0.1753^c^
Mean (SD)	79.53 (80.94)	115.63 (108.68)	​
Range	0.62–382.62	7.43–302.63	​
Median	56.20	51.08	​
95%CI	[59.79; 99.27]	[55.45; 175.82]	​
PCT (ng/mL)	​	​	**0.0016** ^c^
Mean (SD)	1.23 (6.00)	3.08 (8.78)	​
Range	0.01–37.58	0.04–29.52	​
Median	0.09	0.39	​
95%CI	[−0.69; 3.15]	[−2.82; 8.97]	​
LDH (U/L)	​	​	**0.0360** ^c^
Mean (SD)	347.07 (205.01)	563.60 (410.62)	​
Range	105.00–1,170.00	163.00–1,546.00	​
Median	279.00	395.50	​
95%CI	[286.18; 407.95]	[269.86; 857.34]	​
Lymphocytes (×10^9^/L)	​	​	0.0851^c^
Mean (SD)	1.09 (0.52)	0.86 (0.38)	​
Range	0.38–3.31	0.30–1.56	​
Median	1.00	0.72	​
95%CI	[0.96; 1.22]	[0.64; 1.07]	​
BMI (kg/m^2^)	​	​	0.0910^c^
<18.5	1 (1.5%)	2 (13.3%)	​
18.5–24.99	17 (25.8%)	4 (26.7%)	​
25–29.99	22 (33.3%)	6 (40.0%)	​
30–34.99	15 (22.7%)	3 (20.0%)	​
35–39.99	8 (12.1%)	0 (0.0%)	​
>40	3 (4.5%)	0 (0.0%)	​

^a^ Chi2.

^b^ t-Student.

^c^ U Mann-Whitney.

CRP–C-reactive protein; PCT, procalcitonin; LDH, lactate dehydrogenase; BMI, body mass index; CI, confidence interval; SD, standard deviation. P-values were calculated using the Student’s t-test or Mann–Whitney U test for continuous variables and the χ^2^ test for categorical variables.

Bold values indicate statistically significant results (p < 0.05).

The ROC analysis revealed that C-reactive protein (CRP), procalcitonin (PCT), and lactate dehydrogenase (LDH) were statistically significant predictors of the need for oxygen therapy. CRP demonstrated the strongest discriminative ability, with an AUC of 0.758 (p < 0.0001) and an optimal cut-off point of 20.70 mg/L. PCT and LDH also showed significant predictive value, with AUCs of 0.717 (p = 0.0059) and 0.727 (p = 0.0058), and cut-off points of 0.04 ng/mL and 193 U/L, respectively.

Patients with CRP levels above 20.70 mg/L, PCT levels above 0.04 ng/mL, or LDH levels above 193 U/L were more likely to require oxygen supplementation during hospitalization.

Although the calculated sensitivities were moderate to high, the specificities were relatively low.

Moreover, pairwise comparisons of ROC curves demonstrated that CRP was a statistically superior classifier compared to both PCT and LDH (p < 0.05), indicating its higher accuracy in distinguishing between patients requiring and not requiring oxygen therapy.

A detailed summary of the ROC parameters is presented in Table 4.

**TABLE 4:** Receiver operating characteristic (ROC) analysis of age, tryptase, CRP, PCT, LDH, and lymphocyte count for predicting the need for oxygen therapy in COVID-19 patients.

Predictor	AUC (95%CI)	Sensitivity	Specificity	Cut-off point	*P*-value
Age (years)	0.591 (0.431; 0.750)	93.4%	30.0%	47	0.2668
Tryptase (µg/L)	0.580 (0.430; 0.729)	100.0%	10.0%	1.28	0.2972
CRP (mg/L)	0.758 (0.638; 0.878)	86.9%	60.0%	20.70	**<0.0001**
PCT (ng/mL)	0.717 (0.562; 0.872)	97.4%	16.7%	0.04	**0.0059**
LDH (U/L)	0.727 (0.566; 0.888)	92.9%	30.8%	193	**0.0058**
Lymphocytes (×10^9^/L)	0.611 (0.460; 0.762)	98.3%	21.1%	1.56	0.1480

AUC, area under the curve; CRP–C-reactive protein; PCT, procalcitonin; LDH, lactate dehydrogenase; CI, confidence interval.

Bold values indicate statistically significant results (p < 0.05).

The ROC analysis demonstrated that age, procalcitonin (PCT), and lactate dehydrogenase (LDH) were statistically significant predictors of in-hospital mortality. Age showed moderate predictive ability (AUC = 0.736), while PCT showed the highest discriminative power (AUC = 0.814). LDH also demonstrated acceptable prognostic value with an AUC of 0.714.

The optimal cut-off points for age, PCT, and LDH were 84 years, 0.43 ng/mL, and 814 U/L, respectively. Although the sensitivities were modest, all three parameters achieved high specificities exceeding 90%.

In contrast, tryptase, CRP, and lymphocyte count did not show significant discriminative performance (AUC <0.7; p > 0.05).

Comparative analysis revealed no statistically significant differences between the AUC values of age, PCT, and LDH (p > 0.05), indicating similar predictive accuracies.

Detailed ROC metrics are summarized in Table 5.

**TABLE 5:** Receiver operating characteristic (ROC) analysis of age, tryptase, CRP, PCT, LDH, and lymphocyte count for predicting in-hospital mortality.

Predictor	AUC (95%CI)	Sensitivity	Specificity	Cut-off point	*P*-value
Age (years)	0.736 (0.603; 0.869)	46.7%	91.0%	84	**0.0005**
Tryptase (µg/L)	0.538 (0.391; 0.686)	6.7%	97.0%	21.90	0.6103
CRP (mg/L)	0.613 (0.452; 0.774)	26.7%	95.5%	238.47	0.1686
PCT (ng/mL)	0.814 (0.659; 0.968)	45.5%	95.0%	0.43	**0.0001**
LDH (U/L)	0.714 (0.533; 0.895)	30.0%	97.8%	814	**0.0202**
Lymphocytes (×10^9^/L)	0.352 (0.184; 0.520)	0.0%	98.5%	3.31	0.0844

AUC, area under the curve; CRP–C-reactive protein; PCT, procalcitonin; LDH, lactate dehydrogenase; CI, confidence interval.

Bold values indicate statistically significant results (p < 0.05).

The odds ratio analysis identified age over 65 years (OR = 5.60, p = 0.0155), BMI below 18.5 (OR = 10.00, p = 0.0340), and PCT above 0.1 ng/mL (OR = 15.00, p = 0.0068) as statistically significant predictors of in-hospital mortality among COVID-19 patients. Other factors, including gender, hospitalization duration, tryptase levels, CRP, LDH, and lymphocyte counts, did not show statistically significant associations with mortality. The results of the univariate odds ratio analysis for predictors of in-hospital mortality are presented in Table 6.

**TABLE 6:** Odds ratio (OR) analysis for predictors of in-hospital mortality in COVID-19 patients.

Gender	OR	95%CI	*P*-value
F	1.12	[0.356, 3.521]	0.4231
M	0.89	[0.284, 2.807]	0.4231
Age (years)			
<65 lat	0.18	[0.037, 0.854]	**0.0155**
>65 lat	5.60	[1.171, 26.749]	**0.0155**
Hospitalization duration (days)			
1 week	0.68	[0.172, 2.693]	0.2917
2 weeks	1.12	[0.356, 3.521]	0.4231
More than 1 week	1.47	[0.371, 5.815]	0.2917
More than 2 weeks	1.19	[0.379, 3.762]	0.3807
Tryptase (µg/L)			
Norm	0.90	[0.094, 8.714]	0.4649
Elevated	1.11	[0.115, 10.682]	0.4649
BMI (kg/m^2^)			
<18.5	10	[0.843, 118.605]	**0.0340**
18.5–24.99	1.05	[0.294, 3.734]	0.4711
25–29.99	1.33	[0.421, 4.223]	0.3124
30–34.99	0.85	[0.212, 3.413]	0.4094
35–39.99	0.22	[0.012, 4.062]	0.3102
>40	0.59	[0.029, 11.930]	0.7276
CRP (mg/L)			
<5	0.37	[0.019, 6.990]	0.5046
>5	2.73	[0.162, 12.981]	0.5046
PCT (ng/mL)			
<0,1	0.07	[0.008, 0.573]	**0.0068**
>0,1	15.00	[1.746, 128.870]	**0.0068**
LDH (U/L)			
<240	0.19	[0.022, 1.629]	0.0648
>240	5.28	[0.614, 45.337]	0.0648
Lymphocytes (×10^9^/L)			
​	​	​	​
<1	2.50	[0.712, 8.778]	0.0764
1–4.5	0.40	[0.114, 1.404]	0.0764

OR, odds ratio; CI, confidence interval; CRP–C-reactive protein; PCT, procalcitonin; LDH, lactate dehydrogenase; BMI, body mass index.

Bold values indicate statistically significant results (p < 0.05).

The median hospital stay did not differ between groups, and no significant associations were found between tryptase levels and either length of hospitalization or the occurrence of complications.

## Discussion

The results of our study confirm the prognostic relevance of classical inflammatory markers in COVID-19 and underscore the limited utility of serum tryptase measured at admission. Elevated CRP, PCT, and LDH were strongly associated with severity and mortality in our cohort, consistent with multiple meta-analyses and large observational studies (Hariyanto et al., 2020; Henry et al., 2020; Kumar et al., 2022; Stringer et al., 2021). For instance, Henry et al. demonstrated early in the pandemic that high CRP and LDH were robust predictors of mortality (Henry et al., 2020), and Kumar et al. confirmed that procalcitonin retained independent prognostic value (Kumar et al., 2022). Our results reinforce the clinical utility of these inexpensive and widely available biomarkers for early risk stratification.

By contrast, serum tryptase did not discriminate outcomes in our study. Although tryptase is a biologically plausible marker of mast cell activation and may play a mechanistic role in COVID-19–related inflammation, its clinical utility as a circulating biomarker appears limited. This may reflect the short biological half-life of mature tryptase (approximately 2 hours) and the fact that mast cell activation in COVID-19 is largely tissue-restricted, with minimal release of tryptase into the circulation. Consequently, transient local increases in tryptase may not be captured in single-timepoint serum measurements obtained at hospital admission. It is therefore possible that tryptase could serve as a valuable prognostic marker if measured at multiple time points, which might help to identify the optimal sampling window for detecting transient increases associated with disease progression.

This echoes findings from Tan et al., who showed that chymase, not tryptase, was the mast cell protease most consistently elevated in severe COVID-19 (Tan et al., 2023). Krysko et al. reported increases in tryptase, chymase, and CPA3 correlating with severity, though the effect size for tryptase was smaller. In contrast to the studies by Tan et al. and Krysko et al. which primarily investigated patients with severe or fatal COVID-19—and, in part, post-mortem tissue samples—our cohort included a broader clinical spectrum ranging from mild to severe cases, with serum samples collected exclusively at hospital admission. In the study by Tan et al., key observations were derived mainly from animal models and histopathological analyses demonstrating extensive mast cell activation within the lungs during severe infection. Similarly, Krysko et al. reported increased numbers of alveolar mast cells and elevated levels of mast cell proteases (chymase, CPA3, and tryptase) in patients with severe disease or post-mortem specimens. These differences in disease severity and sampling timing may partly explain why serum tryptase in our study did not show prognostic significance despite its mechanistic relevance in severe COVID-19.

Several pathophysiological considerations support this conclusion. Tryptase has a short half-life (∼2 h), so single-admission samples are unlikely to capture transient surges (Valent et al., 2023). Moreover, mast cell activation in COVID-19 appears to be predominantly tissue-restricted. Autopsy studies demonstrate increased numbers of tryptase+ and chymase + mast cells in COVID-19 lungs, with evidence of degranulation particularly in areas of thrombosis, angiogenesis, and fibrosis (Ackermann et al., 2020; Wismans et al., 2023; Meneses-Preza et al., 2024). This aligns with the vascular pathology described by Ackermann et al., who identified endothelialitis, widespread thrombosis, and intussusceptive angiogenesis as defining features of fatal COVID-19 (Ackermann et al., 2020). Thus, mast cell activity may be highly relevant at the local tissue level but may not be reflected adequately in peripheral blood tryptase.

The discrepancy between tissue pathology and circulating tryptase also highlights the potential for biased mediator release. Mast cells can secrete specific subsets of mediators—including cytokines, chemokines, and lipid mediators—without concomitant release of tryptase, a phenomenon described as “piecemeal degranulation” (Molino et al., 1997; Cicala and Cirino, 2002; Itoh et al., 2005). Indeed, experimental data indicate that SARS-CoV-2 proteins alone do not directly trigger mast cell degranulation, suggesting that activation is secondary to the cytokine milieu and tissue injury (Krysko et al., 2022). This could explain why COVID-19 exhibits clinical and histological features of mast cell activation without corresponding elevations in serum tryptase.

From a mechanistic standpoint, tryptase retains clear pathogenetic relevance. It activates protease-activated receptor-2 (PAR-2) on airway epithelium and endothelium, inducing IL-6 and IL-8 release, barrier dysfunction, and bronchoconstriction (Molino et al., 1997; Cicala and Cirino, 2002; Itoh et al., 2005). It also remodels the extracellular matrix and, together with heparin, modifies fibrin architecture, thereby promoting fibrosis and thrombosis (Prieto-García et al., 2012; Samoszuk et al., 2003). These effects dovetail with the immunothrombotic features of severe COVID-19 and provide a plausible link between mast cell activation and the vascular–fibrotic pathology described in post-mortem lungs (Ackermann et al., 2020; Wismans et al., 2023; Meneses-Preza et al., 2024).

Therapeutically, these insights suggest that mast cell–directed interventions may hold promise. Preclinical work demonstrated that mast cell stabilizers attenuate SARS-CoV-2–induced epithelial inflammation and lung injury (Tan et al., 2023). Observational data have also raised interest in histamine receptor antagonists, including famotidine, which in some studies was associated with improved outcomes, though results remain inconclusive (Freedberg et al., 2020; Janowitz et al., 2022). To translate these insights into clinical practice, better biomarkers are needed. Panels incorporating chymase, CPA3, and prostaglandin D_2_ metabolites, in addition to tryptase, may better capture mast cell activation and identify biomarker-enriched subgroups for targeted therapies.

In summary, our findings support the central role of mast cells in COVID-19 pathogenesis but highlight that serum tryptase alone is not a reliable prognostic marker. Classical inflammatory parameters (CRP, PCT, LDH) remain more informative for clinical risk stratification, while future research should focus on composite mast cell signatures and carefully timed sampling to guide personalized therapeutic approaches.

### Limitations

Several limitations of our study should be acknowledged, although they also highlight directions for future research. The analysis was performed in a single center with a moderate sample size, which provided the advantage of standardized protocols for patient management and laboratory testing but may limit generalizability. Serum tryptase was measured only at admission; while this design reflects real-world clinical practice, it may underestimate transient peaks due to the short half-life of mature tryptase. On the other hand, this approach strengthens the relevance of our findings for routine hospital workflows, where single-timepoint measurements are the norm.

### Conclusion

In summary, our findings suggest that serum tryptase measured at hospital admission has limited prognostic value in COVID-19 but may still reflect aspects of mast cell involvement in disease pathogenesis. Identifying the optimal sampling window for detecting transient increases associated with disease progression is crucial for further studies. In contrast, established inflammatory markers such as CRP, PCT, and LDH showed stronger associations with severity and mortality. These results indicate that while mast cell activation likely contributes to the immunopathology of COVID-19, systemic tryptase levels alone are insufficient for clinical risk stratification. These negative findings have practical implications, indicating that measuring serum tryptase at admission is unlikely to influence clinical decision-making and should not replace or complement established inflammatory markers. Further studies incorporating longitudinal sampling and multi-marker approaches are warranted to better define the diagnostic and therapeutic relevance of mast cell–related pathways in COVID-19.

## Data Availability

The datasets presented in this study can be found in online repositories. The names of the repository/repositories and accession number(s) can be found below: https://doi.org/10.5281/zenodo.18088869.

## Footnotes

1
http://www.statsoft.com/
